# Education Level and Cardioprotective Dietary Patterns in Polish Post-MI Patients: A Cross-Sectional Study Using the KomPAN Tool

**DOI:** 10.3390/nu17183018

**Published:** 2025-09-22

**Authors:** Elżbieta Szczepańska, Barbara Janota, Karolina Janion, Krzysztof Biernacki, Oskar Kowalski

**Affiliations:** 1 Department of Human Nutrition, Department of Dietetics, Faculty of Health Sciences in Bytom, Medical University of Silesia in Katowice, ul. Jordana 19, 41-808 Zabrze, Poland;eszczepanska@sum.edu.pl (E.S.);; 2 Department of Basic Medical Sciences, Faculty of Public Health in Bytom, Medical University of Silesia in Katowice, 41-902 Bytom, Poland; 3 Silesian College of Medicine in Katowice, ul. Adam Mickiewicza 29, 40-085 Katowice, Poland; 4 Department of Medical and Molecular Biology, Faculty of Medical Sciences in Zabrze, Medical University of Silesia in Katowice, Jordana 19, 41-808 Zabrze, Poland; kbiernacki@sum.edu.pl; 5 Silesian Center for Heart Diseases, ul. Marii Curie-Skłodowskiej 9, 41-800 Zabrze, Poland

**Keywords:** dietary patterns, myocardial infarction, education level

## Abstract

**Background:** Among patients who have experienced a myocardial infarction, adherence to the principles of healthy eating becomes particularly important. These behaviors may potentially depend on the level of education. **Objectives:** The aim of this study is to assess the relationship between the level of education and dietary behaviors potentially beneficial to health among patients hospitalized due to a previous myocardial infarction. **Methods:** This study includes 164 patients of the Silesian Center for Heart Diseases in Zabrze (Poland). The research tool used was the KomPAN questionnaire for assessing dietary beliefs and habits. The analysis focused on the part of the questionnaire related to the consumption of products with potentially beneficial health effects. To assess diet quality and its association with educational level, the pro-Healthy Diet Index (pHDI) was used. **Results:** The participants consumed an average of 3.42 ± 0.81 meals per day, with individuals with higher education consuming more meals daily (3.85 ± 0.78). Daily consumption of vegetables and fruits was most common among patients with higher education (69.23% and 63.16%, respectively), followed by those with secondary (47.37% and 63.16%), vocational (37.93% and 40.74%), and primary education (33.33% and 33.33%). Statistically significant correlations were observed between education level and frequency of consumption of vegetables (rs = 0.25, *p* = 0.001), fruits (rs = 0.24, *p* = 0.003), legumes (rs = 0.21, *p* = 0.009), whole grain bread (rs = 0.23, *p* = 0.006), and coarse groats (rs = 0.24, *p* = 0.002). The dietary patterns of all study groups were characterized by a moderate level of pro-health features (pHDI among all study participants was 49.87 ± 12.40 points). However, a statistically significant correlation was found between education level and the pro-health diet index (rs = 0.24, *p* = 0.002), with this index increasing with higher education levels. **Conclusions:** Dietary behaviors with potentially beneficial health effects among patients hospitalized due to a myocardial infarction may be related to education level. A higher level of education in our study is associated with more favorable dietary choices compared to a lower level of education.

## 1. Introduction

Education is one of the key socio-economic determinants influencing health behaviors, health status, and the risk of developing chronic diseases, including cardiovascular disease (CVD) [1,2]. According to current knowledge, individuals with higher educational attainment are more likely to follow dietary recommendations. Their diets are typically characterized by higher intake of vegetables, fruits, whole grains, and fish, accompanied by reduced consumption of simple sugars, saturated fats, and highly processed foods [3,4]. Consequently, a high level of education is often associated with a lower prevalence of atherosclerosis risk factors and ischemic heart disease.

In populations of patients with a history of myocardial infarction (MI), adherence to healthy eating principles becomes particularly significant. Secondary prevention—implemented both in hospital and outpatient settings—should include lifestyle modification, particularly dietary improvement, which can substantially reduce the risk of recurrent cardiac events and mortality [5,6]. The guidelines of the European Society of Cardiology (ESC) therefore recommend, among others, the Mediterranean and DASH diets as pro-health dietary patterns [4].

The relationship between education level and dietary habits is complex and influenced by multiple factors. These include food availability, income, cultural beliefs, and the level of health literacy—that is, the ability to understand, evaluate, and apply health and nutrition-related information in daily life [2,7]. Studies suggest that nutritional interventions are most beneficial among individuals with lower levels of education, although this group often faces the greatest barriers in accessing reliable health education, including dietary guidance [8]. It is therefore important to determine whether inadequate dietary patterns and behaviors may be a consequence of low educational attainment, and whether greater attention should be given to improving access to health education.

As previously indicated, existing research confirms the association between education and diet quality. However, data on this relationship in populations of patients after acute cardiovascular events are lacking. Meanwhile, according to Fuller et al., deterioration in health status within a year after a myocardial infarction is particularly pronounced among patients with lower education levels [9]. A study conducted in Indonesia demonstrated that individuals with higher education consumed, on average, 30% more health-promoting foods than those with lower education levels [10]. Similarly, analyses from China showed that a higher education level was associated with greater likelihood of post-MI lifestyle changes, including dietary improvements [11]. However, in relation to Polish patients hospitalized due to myocardial infarction, there is a lack of data on whether such associations exist.

To assess patients’ dietary beliefs and habits, standardized tools are often used, such as the Beliefs and Eating Habits Questionnaire (KomPAN), developed by the Committee of Human Nutrition Science of the Polish Academy of Sciences (PAS). This instrument allows for the evaluation of frequency of consumption of foods that are beneficial or detrimental to health, and enables the calculation of dietary indices, including the pro-healthy diet index (pHDI) [12]. The pHDI has been shown to correlate positively with nutritional knowledge, physical activity, and overall health status [13]. In Poland, the KomPAN questionnaire has been successfully validated and shows good reliability regardless of respondents’ education level, age, or employment status [12]. This means that it provides consistent and comparable results across different social groups, making it suitable for use among post-MI patients.

The aim of this study is to assess the relationship between educational attainment and dietary behaviors potentially beneficial to health among patients hospitalized following myocardial infarction. The pro-healthy diet index (pHDI) and the frequency of consumption of selected food groups were used to determine whether education level may serve as a predictor of health-promoting dietary behaviors, thus supporting the individualization of preventive and educational interventions in clinical practice.

## 2. Materials and Methods

### 2.1. Study Group

This study involved 164 patients of the Silesian Center for Heart Diseases in Zabrze (SCCS), Poland, who were hospitalized due to a recent myocardial infarction (MI), between March 2022 and October 2024. The research was conducted in person, in accordance with the principles of the Declaration of Helsinki. The study protocol was approved by the Bioethics Committee of the Medical University of Silesia in Katowice (Resolution No. PCN/CBN/0022/KB1/91/21, dated 6 July 2021). All participants were informed about the study procedures, and provided written informed consent to participate. Inclusion criteria were (1) age over 18 years, (2) hospitalization due to a recent MI (from day 2 to day 30 post-MI), (3) functional independence and mobility sufficient to perform self-care and ambulate independently, and (4) written informed consent to participate. Exclusion criteria included (1) complicated MI course, (2) inability to move or perform self-care independently, (3) impaired psychosocial functioning preventing independent response to the questionnaire, and (4) lack of consent to participate. The participants filled out the questionnaire based on their dietary habits prior to the myocardial infarction (MI) as soon as their health status allowed. The overview of Polish educational levels and their corresponding International Standard Classification of Education (ISCED) levels are presented in Supplementary Table S1.

### 2.2. Demografic Data

The questionnaire collected data on the following sociodemographic and descriptive variables: sex; year of birth; self-reported body mass (in kilograms) and height (in centimeters); size of the residential area (options: rural area, a city with <20,000 inhabitants, a city with 20,000–100,000 inhabitants, or a city with >100,000 inhabitants); family size (number of members living in the household); self-assessed personal financial situation (options: below average, average, or above average); self-assessed household financial situation (options: very poor, poor, average, good, or very good); and the highest level of education achieved (options: primary, vocational, secondary, or higher). Biometric data (weight, height) were additionally verified on site.

The questionnaire also assessed dietary habits, including the number of main meals consumed on a typical day (excluding snacks), and the frequency of eating out (options: never, 1–3 times per month, once a week, several times per week, once a day, or several times a day). Body Mass Index (BMI) was calculated using the verified self-reported body mass and height for each patient. A detailed breakdown of responses to these demographic questions for the entire study group and by education level is provided in Supplementary Table S2, while participants’ sex, age, and calculated BMI are presented in Table 1.

### 2.3. Research Tool and Research Procedures

The research tool was the KomPAN questionnaire (Questionnaire for the Assessment of Dietary Views and Habits), which evaluates dietary patterns during the year preceding the study [14]. The questionnaire consists of four main sections, including: dietary habits (e.g., number of meals, meal regularity, snacking, use of salt and sugar), frequency of food consumption (including foods potentially beneficial and harmful to health), lifestyle (e.g., alcohol consumption, smoking, sleep, and physical activity), and sociodemographic data.

This study utilized the section focused on the consumption of food products with potentially beneficial health effects.

The frequency of consumption of individual food products was assessed using the following scale: never, 1–3 times per month, once a week, several times per week, once a day, several times a day.

To assess diet quality and its association with educational level, the pro-Healthy Diet Index (pHDI) was used. This index includes ten food groups considered potentially beneficial to health: whole grain bread, whole grain groats and pasta, milk, fermented milk beverages, cottage cheese, white meat dishes, fish, legume-based dishes, fruits, and vegetables.

The pHDI was calculated by summing the frequencies of consumption of these ten food groups, assigning scores as follows: never = 0, 1–3 times per month = 1, once a week = 2, several times per week = 3, once a day = 4, several times a day = 5, and normalizing the result to 0–100 points range. In case of missing patient answer the normalization was performed using the patients maximum possible sum (e.g., 45 instead of 50 with 9 answers). The validity of the used dietary scale performed using the Cronbach’s Alpha (α = 0.68) may suggest a questionable scale, however due to standardized nature of KomPAN questionnaire α value lower than 0.7, but very close to it may reflect the multidimensional nature of dietary patterns. To further validate the questionnaire scale we calculated the McDonald’s Omega total (ωt = 0.79) which indicates a good overall reliability of the used scales.

Calculation formula:

pHDI (points) = (100/(maximum sum of frequency scores for 10 food groups)) × sum of frequency scores for 10 food groups.

pHDI range: 0–100.

Interpretation of pHDI (intensity of health-promoting dietary characteristics) [14]:

Low: 0–33 points.

Moderate: 34–66 points.

High: 67–100 points.

### 2.4. Statistical Analysis and Data Handling

All questionnaire data upon receiving have been inputted into the Microsoft Excel file, and pHDI calculations have been performed. Missing data for patients have been excluded from the analysis by the R function’s default data handling. There are no missing values regarding age, sex, BMI, or education levels for all 164 patients.

Statistical analysis and data visualization were performed using R version 4.5.0 (The R Foundation for Statistical Computing, Vienna, Austria) in RStudio version 2024.12.1 build 563 (PBC, Boston, MA, USA), with the stats package (version 4.5.0). Comparisons of frequency of responses and demographic characteristics across groups defined by educational level were performed using the Kruskal–Wallis test. Percentages refer to the number of participants within each education group who responded to the given item. Demographic data in tables are presented as mean ± standard deviation and median with interquartile range (IQR) for questionary data. Spearman’s rank correlation coefficient (r_s_) was used to assess the correlation between frequency of consumption of selected food groups and demographic variables among the studied patients. Calculation of Cronbach’s Alpha and McDonald’s Omega to validate questionnaire scales were performed using psych (version 2.5.6) package. A multiple linear regression model was conducted to identify the key demographic and situational parameters associated with pHDI with the use of tidyvers (version 2.0.0) package. A backward stepwise approach was adapted, starting with a full model that included all parameters and removing variables that were not statistically significant (*p* > 0.1) and did not contributed significantly to the overall model fit as measured by the Adjusted R-square.

### 2.5. Visualization and Clustering

**Figure 1:**
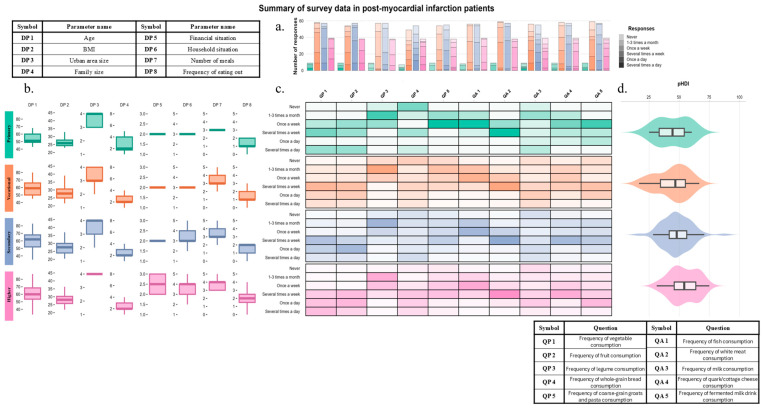
Eating habits in patient groups, according to education level (primary—green, vocational—orange, secondary—blue, higher—pink). Demographic parameter (DP1–DP8) symbols are explained in the top left corner table, and questions concerning the frequency of plant consumption (QP1–QP5) and animal products (QA1–QA5) are explained in the bottom left table. (**a**) Cumulative bar charts illustrating the quantitative distribution of responses to individual questions within specific patient groups. (**b**) Distribution of values for demographic data, as well as the number of meals and frequency of eating out (boxplots). The bolded central value represents the median, the box boundaries indicate the 1st and 3rd quartiles, and the whiskers represent 1.5 times the interquartile range (IQR). (**c**) Percentage frequency of responses to the questions (central heatmap). The darker the coloration, the higher the frequency of responses. (**d**) Distribution of pHDI values across study groups (violin plots). These are boxplots are combined with kernel density estimates. The bolded central line represents the median, the box boundaries denote the 1st and 3rd quartiles, and the whiskers represent 1.5 times the interquartile range (IQR).

Comprehensive data visualization was conducted using R version 4.5.0 (The R Foundation for Statistical Computing, Vienna, Austria) in RStudio version 2024.12.1 build 563 (PBC, Boston, MA, USA) [15], utilizing a suite of integrated packages to prepare the data for analysis, which included using dplyr (version 1.1.4) [16] for data filtering and transformation, tidyr (version 1.3.1) [17] for structuring the data into a clean, tidy format, stringr (version 1.5.1) [18] for processing character strings, and purrr (version 1.0.4) [19] for functional programming to streamline repetitive tasks. Radar/spider plots were generated using the fmsb (version 0.7.6) package [20]. All graphic elements of the summary plot were created using ggplot2 (version 3.5.2) [21] and ggh4x (version 0.3.0) [22]. Hierarchical clustering based on Spearman correlation values between frequency of food group consumption and demographic variables was performed using Euclidean distance calculations and visualized as dendrograms with the ggdendro (version 0.2.0) package [23]. The final multi-panel figure was assembled using the patchwork (version 1.3.0) package [24]. In multi panel figure color coding for education level groups have been used. Primary education in green, vocational in orange, secondary in blue and higher in pink. Additional tables containing description for abbreviations of demographic parameters (DP) and questions concerning frequency of consumption of plant products (QP) and animal products (QA) have been added for quick interpretation. The panels have been aligned to the central Figure 1c, to align the questions responses (between Figure 1a,c) and align the educational group (between Figure 1b–d).

Portions of the text were translated from Polish into English using the ChatGPT language model (OpenAI, July 2025 version). The translation was subsequently manually edited by the author.

## 3. Results

### 3.1. Characteristics of the Study Grup

This study included 164 patients, comprising 122 men (74.39%) and 42 women (25.61%); a detailed breakdown of the sociodemographic characteristics is provided in Table 1. Primary education was reported by 5.48% (*n* = 9) of participants, vocational education by 35.97% (*n* = 59), secondary education by 34.75% (*n* = 57), and higher education by 23.78% (*n* = 39). The mean Body Mass Index (BMI) of all participants was 27.95 ± 4.33 kg/m^2^. The mean BMI by education level was 26.40 ± 3.12 kg/m^2^ (primary), 28.19 ± 4.15 kg/m^2^ (vocational), 27.52 ± 4.26 kg/m^2^ (secondary), and 28.57 ± 4.91 kg/m^2^ (higher).

No statistically significant differences were found between education groups in terms of sex (*p* = 0.083), age (*p* = 0.423), BMI (*p* = 0.502), and family size (*p* = 0.831) (Figure 1b).

### 3.2. Dietary Behaviors

Participants consumed an average of 3.42 ± 0.81 meals per day. Individuals with higher education consumed significantly more meals daily (3.85 ± 0.78) than those with secondary (3.30 ± 0.87, *p* = 0.003) or vocational education (3.25 ± 0.98, *p* < 0.001). Those with primary education consumed 3.44 ± 0.88 meals per day on average, which did not significantly differ from the other groups. A total of 55.56% of individuals with primary education reported not eating meals outside the home, compared to 57.63% with vocational education, 45.61% with secondary education, and only 20.51% with higher education. Educational level was weakly but significantly correlated with the total number of meals consumed daily (rs = 0.24, *p* = 0.002) and the frequency of eating meals outside the home (rs = 0.29, *p* < 0.001) (Figure 1b).

Analysis of the results revealed that 48.47% and 53.80% of all participants consumed vegetables and fruits, respectively, at least once daily. At least once weekly consumption of legumes was most common among those with higher education (50%). Whole grain bread was least consumed by participants with primary education (42.86%). Similarly, the lowest consumption of coarse groats was reported among vocational (18.52%) education group (Table 2, Figure 1a,c).

Statistically significant correlations were observed between education level and the frequency of consumption of vegetables (rs = 0.25, *p* = 0.001), fruits (rs = 0.24, *p* = 0.003), legumes (rs = 0.21, *p* = 0.009), whole grain bread (rs = 0.23, *p* = 0.006), and coarse groats (rs = 0.24, *p* = 0.002). A higher level of education was associated with more frequent consumption of these food groups.

In total, 65.41% of the participants reported consuming fish at least once per week. The highest weekly fish consumption was noted among those with primary education (75.00%). White meat was consumed daily by 5.52% of participants—most commonly among those with higher education (10.5%). None of the participants with primary education reported daily consumption of white meat. Regarding dairy product consumption, daily intake was reported for milk (39.62%, most frequently among those with vocational education—42.86%), fermented milk beverages (29.27%, most frequently among those with higher education—43.59%), and cottage cheese (11.66%, most frequently among those with vocational education—13.79%) (Table 3, Figure 1a,c).

The mean pro-Healthy Diet Index (pHDI) among all study participants was 49.87 ± 12.40 points. In subgroups stratified by education, the values were as follows: 46.87 ± 12.58 (primary education), 46.40 ± 13.03 (vocational), 50.00 ± 11.47 (secondary), and 55.61 ± 10.87 points (higher education). These results indicate that the dietary patterns of all groups demonstrated a moderate level of health-promoting characteristics (Table 4). A statistically significant correlation was found between education level and pHDI (rs = 0.24, *p* = 0.002), with higher education being associated with a significantly higher pHDI score.

Furthermore, pHDI was significantly higher among participants with higher education (mean pHDI = 55.61 ± 10.87) compared to those with secondary education (mean pHDI = 50.00 ± 11.47, *p* = 0.018) and vocational education (mean pHDI = 46.40 ± 13.03, *p* = 0.002) (Figure 1d).

Across all education groups, a similar profile of food group consumption frequency was observed (Figure 1c). However, the pHDI value among participants with higher education was clearly elevated. This increase was most notable when comparing the vocational, secondary, and higher education groups.

To identify the most influential predictors of pHDI, a hierarchical linear regression analysis was conducted (Table 5). The initial full model, including all ten variables, was statistically significant (F(10, 138) = 3.018, *p* = 0.002) and explained 12.0% of the variance in pHDI (adjusted R^2^ = 0.120).

Following a backward elimination procedure, a ‘best fit’ model was identified that maximized the explained variance (Adjusted R^2^ = 0.129). This model (Table 5, Model 2) remained significant overall (*p* < 0.001) and indicated that education, age, and frequency of eating out were significant predictors. Notably, family size, which was significant in the full model, lost its significance, highlighting the interdependence among predictors.

To derive the most parsimonious model, the elimination process was continued until only significant variables remained. The final model (*p* < 0.001) retained education as the sole significant predictor. This model indicates that for every one-unit increase in education, pHDI is predicted to increase by 3.85 units (β = 3.85, *p* < 0.001), with the model accounting for 6.8% of the total variance in pHDI.

Cluster analysis based on correlation coefficients showed that, in terms of correlation profiles between food consumption frequency and demographic data, the groups with primary and higher education were the most similar. However, due to the relatively small sample size, these findings require further investigation (Supplementary Figure S1a,b).

Similarities in the correlation profiles (Supplementary Figure S1b) between demographic parameters may suggest the presence of dietary patterns influenced by financial circumstances (both individual and household), particularly in groups with primary, vocational, and higher education (Supplementary Figure S1a). In vocational, higher, and secondary education groups, similarities were observed in the correlation profile between BMI and other demographic parameters, especially the number of household members. In contrast, among individuals with primary education, the correlation profile for BMI differed from other parameters.

## 4. Discussion

CVD requires structured dietary therapy, while its prevention should be based on adherence to the principles of rational and balanced nutrition [25]. The present study aimed to examine whether there are associations between the level of education among patients who experienced myocardial infarction (MI) and their health-promoting dietary behaviors. The primary recommended dietary pattern is the Mediterranean diet, which is advised both for the primary and secondary prevention of CVD [26]. This diet emphasizes the regular consumption of foods with high antioxidant potential, including vegetables, fruits, legumes, plant oils, fish, and white meat [27]. At the same time, it recommends the elimination of pro-oxidative foods, such as highly processed products low in fiber and rich in saturated fats and simple sugars [27,28]. The DASH (Dietary Approaches to Stop Hypertension) diet, similar to the Mediterranean diet, places even more emphasis on reducing salt and alcohol intake. Like the Mediterranean model, it encourages a high intake of fiber-rich vegetables and fruits, which may reduce, among other things, the risk of hypertension. Additionally, it highlights the importance of whole grain products and the limitation of processed meats and saturated fats [29,30,31].

The principles of the above-mentioned diets align with nutritional guidelines issued by organizations such as the American Heart Association (AHA), the European Society of Cardiology (ESC), and Poland’s National Center for Nutritional Education (NCEZ), targeting individuals aiming to restore and maintain cardiovascular health and prevent CVD [32,33].

The implementation of these recommendations may depend on multiple factors, amongst which socio-economic status—particularly educational attainment—plays a key role. Roszkowska et al., analyzing data from 2012 to 2019, observed more frequent hospitalizations due to CVD among individuals living in regions characterized by low socio-economic development and poor healthcare infrastructure [34]. Similarly, Jamiołkowski et al. assessed the relationship between socio-economic status (SES) and CVD mortality across 66 subregions in Poland, finding that mortality reduction could be achieved through education, poverty alleviation, and employment, especially in underdeveloped areas [35].

According to available research, securing employment—thereby reducing poverty—often correlates with higher education. Individuals with higher levels of education are more likely to find and retain employment, learn new skills, and earn more throughout their working life compared to those with lower education levels. Health outcomes are also better among the more educated, who are statistically more likely to live longer and in better health [36].

Research by various authors supports the existence of such relationships. For instance, McCullough et al., in a study involving Americans, demonstrated that better diet quality was associated with higher income and educational attainment [37]. Similarly, Poklar et al., who assessed adherence to the Mediterranean diet in relation to socio-demographic factors in Slovenia, found low adherence overall, but noted strong associations with education and place of residence [38].

Our findings reveal correlations between education level and the frequency of consumption of vegetables, fruits, and legumes among patients. Similar results were obtained by Górska-Warsewicz et al., who showed that, in Poland, the amount of vegetables and potatoes consumed (including processed forms) was influenced by education level, income, and socio-economic status [39]. Stea et al., in a study evaluating fruit and vegetable consumption in 21 European countries, also found that higher education was associated with increased intake of these food groups [40].

Gomez et al., analyzing the impact of SES—including education, occupation, and income—on diet quality and BMI in Latin American countries, demonstrated that individuals with low SES consumed fewer fruits, vegetables, whole grains, fish, and seafood, and more red and processed meats compared to those with high SES. Furthermore, diet quality improved as SES increased. Interestingly, BMI did not differ significantly across SES groups, averaging around 27 kg/m^2^. However, women had significantly higher BMI than men within the low- and medium-SES groups, but not in the high-SES group [41].

Ahmadi Tabatabai et al., investigating factors influencing fruit and vegetable consumption among diabetic patients, found that education, employment, and income were significantly associated with their intake [42]. Our study showed that regardless of education level, the frequency of vegetable, fruit, and legume consumption was insufficient. Similarly, Mrazova et al., in a study of cardiovascular risk factors among men hospitalized after myocardial infarction, observed adverse dietary behaviors [43].

Numerous studies have shown that plant-based diets are associated with positive cardiovascular effects due to the presence of protective components such as dietary fiber and antioxidants, and reduced levels of saturated fats [29,43,44].

A healthy diet is a cornerstone of both primary and secondary CVD prevention. Optimal nutritional strategies reduce the risk of diabetes, hypertension, and stroke, help prevent obesity, and consequently lower CVD risk. Nutrition should play a central role in CVD prevention as a non-pharmacological factor. More educational strategies are needed to emphasize the critical importance of dietary habits for healthy living and aging [45,46]. Special attention should be given to populations affected by socio-economic inequalities, including disparities in education, income, and occupation, through campaigns promoting adequate diet quality. Public health interventions should aim to reduce the intake of energy-dense foods high in solid fats, added sugars, and sodium, while improving access to healthy eating information.

Policymakers, researchers, and healthcare professionals should collaborate to promote a healthy lifestyle—encouraging balanced diets, limited alcohol consumption, smoking cessation, and increased physical activity [47].

The importance of health and nutrition education is increasingly recognized in Polish educational policy. An example of this is the planned introduction in September 2025 of a new school subject—health education. The main objective of this course is to equip students with the ability to maintain and improve their health in all dimensions (i.e., physical, mental, social, sexual, and environmental), and to ensure the health and safety of themselves and others at all stages of life. The curriculum will include modules such as nutrition, physical activity, and addiction prevention, and will be implemented in primary schools (grades IV–VI and VII–VIII), as well as secondary schools [48].

Such early educational efforts align with the recommendations of international institutions such as the AHA, U.S. Preventive Services Task Force (USPSTF), and ESC, which emphasize that the promotion of healthy dietary habits should begin in school and be supported by the healthcare system [6].

A major strength of this study lies in its innovative approach to examining dietary behaviors in the context of secondary prevention of myocardial infarction, with specific reference to educational attainment. The study also highlights the crucial role of patient education, emphasizing the need for it to be introduced as early as possible in the process of knowledge acquisition. However, this study has limitations. A limitation of the study is the disproportion between the genders of the analyzed persons. In the future, it would be valuable to analyze eating behaviors taking into account other socio-demographic factors, such as finances, place of residence, and origin.

## 5. Conclusions

Dietary behaviors with potentially beneficial effects on health among patients hospitalized due to a history of myocardial infarction (MI) may be associated with their level of education. Patients with higher educational attainment tend to make more favorable dietary choices compared to those with lower education levels, although the frequency of consumption of certain health-promoting foods remains insufficient. More educational strategies should be implemented to emphasize the paramount importance of proper nutrition, particularly in the first levels of education. In addition to structured educational programs, individualized patient-tailored education should be ensured, delivered both in outpatient clinics and hospital wards, in order to improve eating behaviors among people with insufficient knowledge in the field of nutrition.

## Figures and Tables

**Table 1:** Sociodemographic characteristics of the study population.

	Total	Primary	Vocational	Secondary	Higher
	*N* (%)	*N* (%)	*N* (%)	*N* (%)	*N* (%)
Number of patients	167 (100%)	9 (5.4%)	59 (35.9%)	57 (35.3%)	39 (23.4%)
Men	122 (74.4%)	4 (44.4%)	41 (69.5%)	46 (80.7%)	31 (79.5%)
Women	42 (25.6%)	5 (55.6%)	18 (30.5%)	11 (19.3%)	8 (20.5%)
	Mean ± SD	Mean ± SD	Mean ± SD	Mean ± SD	Mean ± SD
Age	59.8 ± 11.2	55.9 ± 11.4	58.9 ± 10	60.7 ± 11.8	60.8 ± 12
BMI	28 ± 4.3	26.4 ± 3.1	28.2 ± 4.1	27.5 ± 4.3	28.6 ± 4.9
pHDI	49.9 ± 12.4	46.9 ± 12.6	46.4 ± 13	50 ± 11.5	55.6 ± 10.9
	Median (IQR)	Median (IQR)	Median (IQR)	Median (IQR)	Median (IQR)
Urban area size	4 (3–4)	4 (3–4)	3 (3–4)	4 (3–4)	4 (4–4)
Family size	2 (2–3)	2 (1.5–4)	2 (2–3)	2 (2–3)	2 (2–3)
Financial situation	2 (2–2)	2 (2–2)	2 (2–2)	2 (2–2)	2.5 (2–3)
Household situation	3 (3–4)	3 (2.75–3)	3 (3–3)	3 (3–4)	4 (3–4)
Number of meals	3 (3–4)	3 (3–3.5)	3 (3–4)	3 (3–4)	4 (3–4)
Frequency of eating out	2 (1–2)	1 (1–2)	1 (1–2)	2 (1–2)	2 (1.25–2.75)
pHDI	50 (42.1–59)	46.67 (35.9–57.5)	48.89 (36.5–57.3)	50 (43–58)	56 (48–65.6)

**Table 2:** Frequency of consumption of selected groups of plant products that prevent heart and vascular diseases depending on education level.

	Education Level	All		Primary		Vocational		Secondary		Higher	
		*n*	%	*n*	%	*n*	%	*n*	%	*n*	%
Frequency of vegetable consumption	Never	1	0.61	0	0.00	1	1.72	0	0.00	0	0.00
	1–3 times a month	10	6.13	0	0.00	6	10.34	4	7.02	0	0.00
	Once a week	10	6.13	2	22.22	5	8.62	2	3.51	1	2.56
	Several times a week	63	38.65	4	44.44	24	41.38	24	42.11	11	28.21
	Once a day	44	26.99	0	0.00	13	22.41	18	31.58	13	33.33
	Several times a day	35	21.47	3	33.33	9	15.52	9	15.79	14	35.90
Frequency of fruit consumption	Never	1	0.63	0	0.00	1	1.85	0	0.00	0	0.00
	1–3 times a month	9	5.70	0	0.00	6	11.11	3	5.26	0	0.00
	Once a week	11	6.96	3	33.33	6	11.11	2	3.51	0	0.00
	Several times a week	52	32.91	3	33.33	19	35.19	16	28.07	14	36.84
	Once a day	57	36.08	0	0.00	15	27.78	27	47.37	15	39.47
	Several times a day	28	17.72	3	33.33	7	12.96	9	15.79	9	23.68
Frequency of legume consumption	Never	18	11.18	1	11.11	10	17.54	6	10.53	1	2.63
	1–3 times a month	82	50.93	5	55.56	30	52.63	29	50.88	18	47.37
	Once a week	46	28.57	3	33.33	15	26.32	15	26.32	13	34.21
	Several times a week	13	8.07	0	0.00	1	1.75	6	10.53	6	15.79
	Once a day	2	1.24	0	0.00	1	1.75	1	1.75	0	0.00
	Several times a day	0	0.00	0	0.00	0	0.00	0	0.00	0	0.00
Frequency of whole-wheat bread consumption	Never	29	19.59	3	42.86	13	26.53	10	18.52	3	7.89
	1–3 times a month	17	11.49	1	14.29	5	10.20	10	18.52	1	2.63
	Once a week	19	12.84	0	0.00	7	14.29	7	12.96	5	13.16
	Several times a week	33	22.30	2	28.57	11	22.45	8	14.81	12	31.58
	Once a day	24	16.22	0	0.00	7	14.29	7	12.96	10	26.32
	Several times a day	26	17.57	1	14.29	6	12.24	12	22.22	7	18.42
Frequency of whole grain groats and pasta consumption	Never	20	12.82	0	0.00	10	18.52	6	10.91	4	10.53
	1–3 times a month	40	25.64	3	33.33	19	35.19	12	21.82	6	15.79
	Once a week	43	27.56	6	66.67	13	24.07	13	23.64	11	28.95
	Several times a week	42	26.92	0	0.00	11	20.37	18	32.73	13	34.21
	Once a day	8	5.13	0	0.00	1	1.85	5	9.09	2	5.26
	Several times a day	3	1.92	0	0.00	0	0.00	1	1.82	2	5.26

% of responses; Supplementary Table S3 contains detailed information concerning missing responses.

**Table 3:** Frequency of consumption of selected groups of animal products that prevent heart and vascular diseases depending on education level.

	Education Level	All		Primary		Vocational		Secondary		Higher	
		*n*	%	*n*	%	*n*	%	*n*	%	*n*	%
Frequency of fish consumption	Never	5	3.14	0	0.00	2	3.57	2	3.57	1	2.56
	1–3 times a month	50	31.45	2	25.00	19	33.93	20	35.71	9	23.08
	Once a week	77	48.43	5	62.50	25	44.64	28	50.00	19	48.72
	Several times a week	23	14.47	1	12.50	9	16.07	4	7.14	9	23.08
	Once a day	4	2.52	0	0.00	1	1.79	2	3.57	1	2.56
	Several times a day	0	0.00	0	0.00	0	0.00	0	0.00	0	0.00
Frequency of white meat consumption	Never	1	0.61	0	0.00	1	1.69	0	0.00	0	0.00
	1–3 times a month	13	7.98	0	0.00	6	10.17	4	7.02	3	7.89
	Once a week	45	27.61	3	33.33	18	30.51	17	29.82	7	18.42
	Several times a week	95	58.28	6	66.67	31	52.54	34	59.65	24	63.16
	Once a day	8	4.91	0	0.00	2	3.39	2	3.51	4	10.53
	Several times a day	1	0.61	0	0.00	1	1.69	0	0.00	0	0.00
Frequency of milk consumption	Never	28	17.61	1	11.11	9	16.07	11	20.00	7	17.95
	1–3 times a month	21	13.21	3	33.33	6	10.71	7	12.73	5	12.82
	Once a week	15	9.43	1	11.11	5	8.93	5	9.09	4	10.26
	Several times a week	32	20.13	1	11.11	12	21.43	10	18.18	9	23.08
	Once a day	39	24.53	1	11.11	15	26.79	12	21.82	11	28.21
	Several times a day	24	15.09	2	22.22	9	16.07	10	18.18	3	7.69
Frequency of cottage cheese consumption	Never	9	5.52	0	0.00	4	6.90	3	5.26	2	5.13
	1–3 times a month	32	19.63	2	22.22	14	24.14	12	21.05	4	10.26
	Once a week	40	24.54	4	44.44	16	27.59	10	17.54	10	25.64
	Several times a week	63	38.65	2	22.22	16	27.59	27	47.37	18	46.15
	Once a day	13	7.98	1	11.11	6	10.34	3	5.26	3	7.69
	Several times a day	6	3.68	0	0.00	2	3.45	2	3.51	2	5.13
Frequency of fermented milk beverages consumption	Never	13	7.93	0	0.00	10	16.95	1	1.75	2	5.13
	1–3 times a month	25	15.24	0	0.00	8	13.56	13	22.81	4	10.26
	Once a week	26	15.85	5	55.56	4	6.78	11	19.30	6	15.38
	Several times a week	52	31.71	2	22.22	20	33.90	20	35.09	10	25.64
	Once a day	44	26.83	2	22.22	16	27.12	11	19.30	15	38.46
	Several times a day	4	2.44	0	0.00	1	1.69	1	1.75	2	5.13

% of responses; Supplementary Table S4 contains detailed information concerning missing responses.

**Table 4:** pHDI levels and classification in the study cohort.

	*n* (%)	Mean ± SD	Low pHDI	Medium pHDI	High pHDI
All	164 (100%)	49.87 ± 12.4	17 (10.37%)	133 (81.1%)	14 (8.54%)
Primary	9 (100%)	46.87 ± 12.58	1 (11.11%)	8 (88.89%)	0 (0%)
Vocational	59 (100%)	46.4 ± 13.03	14 (23.73%)	43 (72.88%)	2 (3.39%)
Secondary	57 (100%)	50 ± 11.47	2 (3.51%)	52 (91.23%)	3 (5.26%)
Higher	39 (100%)	55.61 ± 10.87	0 (0%)	30 (76.92%)	9 (23.08%)

**Table 5:** Hierarchical Multiple Linear Regression models predicting pHDI.

Variable	Model 1 (Full)	Model 2 (Best Fit)	Final Model (Parsimonious)
(Intercept)	21.25 (.)	16.23 (.)	39.20 (*)
Education	3.79 (**)	3.58 (**)	3.85 (*)
Age	0.22 (*)	0.24 (*)	-
Frequency of eating out	3.29 (*)	3.47 (*)	-
Family size	1.82 (*)	1.54 (.)	-
Sex	3.05	3.45	-
Household situation	−1.35	−1.48	-
Number of meals	1.18	1.29	-
Financial situation	−1.07	-	-
BMI	−0.14	-	-
Urban area size	0.44	-	-
Model Fit Statistics			
R-squared	0.179	0.169	0.074
Adjusted R-squared	0.12	0.129	0.068
F-statistic	3.018	4.213	12.98
*p*-value	0.002	<0.001	<0.001

Values are the coefficient estimates (β). Significance codes: (.) *p* < 0.1, (*) *p* < 0.05, (**) *p* < 0.01.

## Data Availability

The data presented in this study are available on request from the corresponding author due to privacy and ethical reasons.

## Supplementary Materials

The following supporting information can be downloaded at: https://www.mdpi.com/article/10.3390/nu17183018/s1, Table S1: Overview of Polish educational levels and their corresponding ISCED levels; Table S2: Sociodemographic questionnaire answers; Table S3: Frequency of consumption of selected groups of plant products that prevent heart and vascular diseases depending on education level; Table S4: Frequency of consumption of selected groups of animal products that prevent heart and vascular diseases depending on education level; Figure S1: Clustering results for Spearman’s correlation coefficient.
