# Supplementary material for: Education Level and Cardioprotective Dietary Patterns in Polish Post-MI Patients: A Cross-Sectional Study Using the KomPAN Tool
Source: Nutrients. 2025 Sep 22;17(18):3018. doi: 10.3390/nu17183018 (PMC12472266; doi:10.3390/nu17183018)
Supplement: Supplementary file 1 [file nutrients-17-03018-s001.zip › nutrients-3817630-supplementary.pdf]

Supplementary Table S1. Overview of Polish educational levels and their corresponding ISCED levels

**Polish educational level**	**ISCED level**	**Qualifications obtained**
Primary education	ISCED 1 + 2	Primary education certificate, Eighth-grader exam
Vocational education	ISCED 3	Vocational certificate/diploma (skilled worker level)
Secondary education	ISCED 3	Maturity certificate, Vocational qualifications
Higher education	ISCED 5-8	Bachelor's, Master's, Doctoral degrees

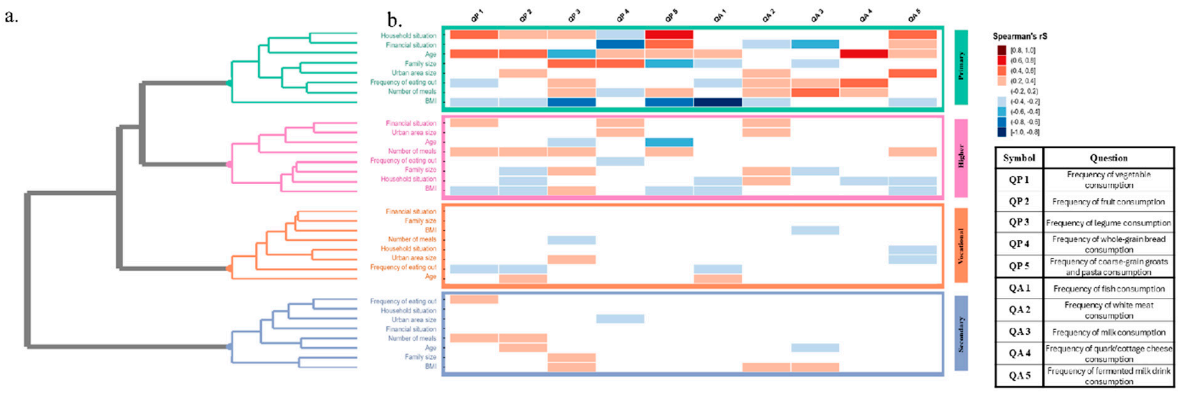

Supplementary Figure S1. Clustering results for Spearman's correlation coefficient

(a) Heatmap with education levels clustered according to their correlation profile within the entire group (gray dendrogram on the left), and demographic parameters ordered according to similarity in correlation profile (colored dendrograms for each education group). (b) Heatmap of correlations between dietary behavior variables (i.e., frequency of consumption of selected food groups) and demographic parameters. Positive Spearman correlation coefficients are marked in red, while negative coefficients are marked in blue.

Supplementary Table S2. Sociodemographic questionnaire answers

Education level		All			Primary			Vocational			Secondary			Higher		
		n	%[a]	%[b]	n	%[a]	%[b]	n	%[a]	%[b]	n	%[a]	%[b]	n	%[a]	%[b]
**Number of main meals**	1 meal	1	0.6	0.6	0	0.0	0.0	1	1.7	1.7	0	0.0	0.0	0	0.0	0.0
	2 meals	8	4.9	4.9	0	0.0	0.0	3	5.1	5.1	5	8.8	8.8	0	0.0	0.0
	3 meals	89	54.3	54.3	7	77.8	77.8	37	62.7	62.7	30	52.6	52.6	15	38.5	38.5
	4 meals	48	29.3	29.3	0	0.0	0.0	16	27.1	27.1	17	29.8	29.8	15	38.5	38.5
	5 and more	17	10.4	10.4	2	22.2	22.2	2	3.4	3.4	4	7.0	7.0	9	23.1	23.1
**Frequency of eating out**	Never	73	44.5	44.5	5	55.6	55.6	34	57.6	57.6	26	45.6	45.6	8	20.5	20.5
	1-3 times a month	72	43.9	43.9	3	33.3	33.3	21	35.6	35.6	29	50.9	50.9	19	48.7	48.7
	Once a week	8	4.9	4.9	0	0.0	0.0	2	3.4	3.4	0	0.0	0.0	6	15.4	15.4
	Several times a week	5	3.0	3.0	0	0.0	0.0	0	0.0	0.0	1	1.8	1.8	4	10.3	10.3
	Once a day	1	0.6	0.6	0	0.0	0.0	1	1.7	1.7	0	0.0	0.0	0	0.0	0.0
	Several times a day	0	0.0	0.0	0	0.0	0.0	0	0.0	0.0	0	0.0	0.0	0	0.0	0.0
**Family size**	No response	6	3.7	3.8	1	11.1	12.5	3	5.1	5.4	1	1.8	1.8	1	2.6	2.6
	1	25	15.2	15.8	2	22.2	25.0	12	20.3	21.4	8	14.0	14.3	3	7.7	7.9
	2	76	46.3	48.1	3	33.3	37.5	22	37.3	39.3	30	52.6	53.6	21	53.8	55.3
	3	33	20.1	20.9	0	0.0	0.0	15	25.4	26.8	11	19.3	19.6	7	17.9	18.4
	4	17	10.4	10.8	2	22.2	25.0	6	10.2	10.7	4	7.0	7.1	5	12.8	13.2
	5	3	1.8	1.9	1	11.1	12.5	0	0.0	0.0	2	3.5	3.6	0	0.0	0.0
	6	2	1.2	1.3	0	0.0	0.0	1	1.7	1.8	0	0.0	0.0	1	2.6	2.6
**Size of the residential area**	No response	4	2.4	2.5	0	0.0	0.0	2	3.4	3.5	2	3.5	3.6	0	0.0	0.0
	Rural area	9	5.5	5.6	0	0.0	0.0	4	6.8	7.0	4	7.0	7.3	1	2.6	2.6
	City with less than 20000 inhabitants	10	6.1	6.3	0	0.0	0.0	7	11.9	12.3	3	5.3	5.5	0	0.0	0.0
	A city with a population between 20000 and 100000	37	22.6	23.1	4	44.4	44.4	19	32.2	33.3	8	14.0	14.5	6	15.4	15.4
	A city with more than 100000 inhabitants	104	63.4	65.0	5	55.6	55.6	27	45.8	47.4	40	70.2	72.7	32	82.1	82.1
**Financial situation**	No response	2	1.2	1.2	0	0.0	0.0	1	1.7	1.7	0	0.0	0.0	1	2.6	2.6
	Below average	8	4.9	4.9	1	11.1	11.1	5	8.5	8.6	2	3.5	3.5	0	0.0	0.0
	Average	123	75.0	75.9	8	88.9	88.9	49	83.1	84.5	47	82.5	82.5	19	48.7	50.0
	Above average	31	18.9	19.1	0	0.0	0.0	4	6.8	6.9	8	14.0	14.0	19	48.7	50.0
**Hou**	No response	5	3.0	3.1	0	0.0	0.0	3	5.1	5.4	1	1.8	1.8	1	2.6	2.6

	Very modestly	2	1.2	1.3	1	11.1	11.1	0	0.0	0.0	1	1.8	1.8	0	0.0	0.0
	Modestly	12	7.3	7.5	1	11.1	11.1	9	15.3	16.1	1	1.8	1.8	1	2.6	2.6
	Normal	81	49.4	50.9	7	77.8	77.8	36	61.0	64.3	28	49.1	50.0	10	25.6	26.3
	Relatively wealthy	57	34.8	35.8	0	0.0	0.0	11	18.6	19.6	24	42.1	42.9	22	56.4	57.9
	Very wealthy	7	4.3	4.4	0	0.0	0.0	0	0.0	0.0	2	3.5	3.6	5	12.8	13.2

[a] % of participants. [b] % of responses

Supplementary Table S3. Frequency of consumption of selected groups of plant products that prevent heart and vascular diseases depending on education level.

	**Education level**	**All (n=164)**			**Primary (n=9)**			**Vocational (n=59)**			**Secondary (n=57)**			**Higher (n=39)**		
		n	%[a]	%[b]	n	%[a]	%[b]	n	%[a]	%[b]	n	%[a]	%[b]	n	%[a]	%[b]
**Frequency of vegetable consumption**	No response	1	0.61	-	0	0.00	-	1	1.69	-	0	0.00	-	0	0.00	-
	Never	1	0.61	0.61	0	0.00	0.00	1	1.69	1.72	0	0.00	0.00	0	0.00	0.00
	1-3 times a month	10	6.10	6.13	0	0.00	0.00	6	10.17	10.34	4	7.02	7.02	0	0.00	0.00
	Once a week	10	6.10	6.13	2	22.22	22.22	5	8.47	8.62	2	3.51	3.51	1	2.56	2.56
	Several times a week	63	38.41	38.65	4	44.44	44.44	24	40.68	41.38	24	42.11	42.11	11	28.21	28.21
	Once a day	44	26.83	26.99	0	0.00	0.00	13	22.03	22.41	18	31.58	31.58	13	33.33	33.33
	Several times a day	35	21.34	21.47	3	33.33	33.33	9	15.25	15.52	9	15.79	15.79	14	35.90	35.90
	No missing responses	163	99.39	100	9	100	100	58	98.31	100	57	100	100	39	100	100
**Frequency of fruit consumption**	No response	6	3.66	-	0	0.00	-	5	8.47	-	0	0.00	-	1	2.56	-
	Never	1	0.61	0.63	0	0.00	0.00	1	1.69	1.85	0	0.00	0.00	0	0.00	0.00
	1-3 times a month	9	5.49	5.70	0	0.00	0.00	6	10.17	11.11	3	5.26	5.26	0	0.00	0.00
	Once a week	11	6.71	6.96	3	33.33	33.33	6	10.17	11.11	2	3.51	3.51	0	0.00	0.00
	Several times a week	52	31.71	32.91	3	33.33	33.33	19	32.20	35.19	16	28.07	28.07	14	35.90	36.84
	Once a day	57	34.76	36.08	0	0.00	0.00	15	25.42	27.78	27	47.37	47.37	15	38.46	39.47
	Several times a day	28	17.07	17.72	3	33.33	33.33	7	11.86	12.96	9	15.79	15.79	9	23.08	23.68
	No missing responses	158	96.34	100	9	100	100	54	91.53	100	57	100	100	38	97.44	100
**Frequency of legume consumption**	No response	3	1.83	-	0	0.00	-	2	3.39	-	0	0.00	-	1	2.56	-
	Never	18	10.98	11.18	1	11.11	11.11	10	16.95	17.54	6	10.53	10.53	1	2.56	2.63
	1-3 times a month	82	50.00	50.93	5	55.56	55.56	30	50.85	52.63	29	50.88	50.88	18	46.15	47.37
	Once a week	46	28.05	28.57	3	33.33	33.33	15	25.42	26.32	15	26.32	26.32	13	33.33	34.21
	Several times a week	13	7.93	8.07	0	0.00	0.00	1	1.69	1.75	6	10.53	10.53	6	15.38	15.79
	Once a day	2	1.22	1.24	0	0.00	0.00	1	1.69	1.75	1	1.75	1.75	0	0.00	0.00
	Several times a day	0	0.00	0.00	0	0.00	0.00	0	0.00	0.00	0	0.00	0.00	0	0.00	0.00
	No missing responses	161	98.17	100	9	100	100	57	96.61	100	57	100	100	38	97.44	100

Frequency of whole-wheat bread consumption	No response	16	9.76	-	2	22.22	-	10	16.95	-	3	5.26	-	1	2.56	-
	Never	29	17.68	19.59	3	33.33	42.86	13	22.03	26.53	10	17.54	18.52	3	7.69	7.89
	1-3 times a month	17	10.37	11.49	1	11.11	14.29	5	8.47	10.20	10	17.54	18.52	1	2.56	2.63
	Once a week	19	11.59	12.84	0	0.00	0.00	7	11.86	14.29	7	12.28	12.96	5	12.82	13.16
	Several times a week	33	20.12	22.30	2	22.22	28.57	11	18.64	22.45	8	14.04	14.81	12	30.77	31.58
	Once a day	24	14.63	16.22	0	0.00	0.00	7	11.86	14.29	7	12.28	12.96	10	25.64	26.32
	Several times a day	26	15.85	17.57	1	11.11	14.29	6	10.17	12.24	12	21.05	22.22	7	17.95	18.42
	No missing responses	148	90.24	100	7	77.78	100	49	83.05	100	54	94.74	100	38	97.44	100
Frequency of whole-grain groats and pasta consumption	No response	8	4.88	-	0	0.00	-	5	8.47	-	2	3.51	-	1	2.56	-
	Never	20	12.20	12.82	0	0.00	0.00	10	16.95	18.52	6	10.53	10.91	4	10.26	10.53
	1-3 times a month	40	24.39	25.64	3	33.33	33.33	19	32.20	35.19	12	21.05	21.82	6	15.38	15.79
	Once a week	43	26.22	27.56	6	66.67	66.67	13	22.03	24.07	13	22.81	23.64	11	28.21	28.95
	Several times a week	42	25.61	26.92	0	0.00	0.00	11	18.64	20.37	18	31.58	32.73	13	33.33	34.21
	Once a day	8	4.88	5.13	0	0.00	0.00	1	1.69	1.85	5	8.77	9.09	2	5.13	5.26
	Several times a day	3	1.83	1.92	0	0.00	0.00	0	0.00	0.00	1	1.75	1.82	2	5.13	5.26
	No missing responses	156	95.12	100	9	100	100	54	91.53	100	55	96.49	100	38	97.44	100

[a] % of participants. [b] % of responses

Supplementary Table S4. Frequency of consumption of selected groups of animal products that prevent heart and vascular diseases depending on education level.

	Education level	All (n=164)			Primary (n=9)			Vocational (n=59)			Secondary (n=57)			Higher (n=39)		
		n	%[a]	%[b]	n	%[a]	%[b]	n	%[a]	%[b]	n	%[a]	%[b]	n	%[a]	%[b]
Frequency of fish consumption	No response	5	3.05	-	1	11.11	-	3	5.08	-	1	1.75	-	0	0.00	-
	Never	5	3.05	3.14	0	0.00	0.00	2	3.39	3.57	2	3.51	3.57	1	2.56	2.56
	1-3 times a month	50	30.49	31.45	2	22.22	25.00	19	32.20	33.93	20	35.09	35.71	9	23.08	23.08
	Once a week	77	46.95	48.43	5	55.56	62.50	25	42.37	44.64	28	49.12	50.00	19	48.72	48.72
	Several times a week	23	14.02	14.47	1	11.11	12.50	9	15.25	16.07	4	7.02	7.14	9	23.08	23.08
	Once a day	4	2.44	2.52	0	0.00	0.00	1	1.69	1.79	2	3.51	3.57	1	2.56	2.56
	Several times a day	0	0.00	0.00	0	0.00	0.00	0	0.00	0.00	0	0.00	0.00	0	0.00	0.00
	No missing responses	159	96.95	100	8	88.89	100	56	94.92	100	56	98.25	100	39	100	100
Frequency of white meat consumption	No response	1	0.61	-	0	0.00	-	0	0.00	-	0	0.00	-	1	2.56	-
	Never	1	0.61	0.61	0	0.00	0.00	1	1.69	1.69	0	0.00	0.00	0	0.00	0.00
	1-3 times a month	13	7.93	7.98	0	0.00	0.00	6	10.17	10.17	4	7.02	7.02	3	7.69	7.89
	Once a week	45	27.44	27.61	3	33.33	33.33	18	30.51	30.51	17	29.82	29.82	7	17.95	18.42
	Several times a week	95	57.93	58.28	6	66.67	66.67	31	52.54	52.54	34	59.65	59.65	24	61.54	63.16
	Once a day	8	4.88	4.91	0	0.00	0.00	2	3.39	3.39	2	3.51	3.51	4	10.26	10.53

	Several times a day	1	0.61	0.61	0	0.00	0.00	1	1.69	1.69	0	0.00	0.00	0	0.00	0.00
	No missing responses	163	99.39	100	9	100	100	59	100	100	57	100	100	38	97.44	100
**Frequency of milk consumption**	No response	5	3.05	-	0	0.00	-	3	5.08	-	2	3.51	-	0	0.00	-
	Never	28	17.07	17.61	1	11.11	11.11	9	15.25	16.07	11	19.30	20.00	7	17.95	17.95
	1-3 times a month	21	12.80	13.21	3	33.33	33.33	6	10.17	10.71	7	12.28	12.73	5	12.82	12.82
	Once a week	15	9.15	9.43	1	11.11	11.11	5	8.47	8.93	5	8.77	9.09	4	10.26	10.26
	Several times a week	32	19.51	20.13	1	11.11	11.11	12	20.34	21.43	10	17.54	18.18	9	23.08	23.08
	Once a day	39	23.78	24.53	1	11.11	11.11	15	25.42	26.79	12	21.05	21.82	11	28.21	28.21
	Several times a day	24	14.63	15.09	2	22.22	22.22	9	15.25	16.07	10	17.54	18.18	3	7.69	7.69
	No missing responses	159	96.95	100	9	100	100	56	94.92	100	55	96.49	100	39	100	100
**Frequency of cottage cheese consumption**	No response	1	0.61	-	0	0.00	-	1	1.69	-	0	0.00	-	0	0.00	-
	Never	9	5.49	5.52	0	0.00	0.00	4	6.78	6.90	3	5.26	5.26	2	5.13	5.13
	1-3 times a month	32	19.51	19.63	2	22.22	22.22	14	23.73	24.14	12	21.05	21.05	4	10.26	10.26
	Once a week	40	24.39	24.54	4	44.44	44.44	16	27.12	27.59	10	17.54	17.54	10	25.64	25.64
	Several times a week	63	38.41	38.65	2	22.22	22.22	16	27.12	27.59	27	47.37	47.37	18	46.15	46.15
	Once a day	13	7.93	7.98	1	11.11	11.11	6	10.17	10.34	3	5.26	5.26	3	7.69	7.69
	Several times a day	6	3.66	3.68	0	0.00	0.00	2	3.39	3.45	2	3.51	3.51	2	5.13	5.13
	No missing responses	163	99.39	100	9	100	100	58	98.31	100	57	100	100	39	100	100
**Frequency of fermented milk beverages consumption**	No response	0	0.00	-	0	0.00	-	0	0.00	-	0	0.00	-	0	0.00	-
	Never	13	7.93	7.93	0	0.00	0.00	10	16.95	16.95	1	1.75	1.75	2	5.13	5.13
	1-3 times a month	25	15.24	15.24	0	0.00	0.00	8	13.56	13.56	13	22.81	22.81	4	10.26	10.26
	Once a week	26	15.85	15.85	5	55.56	55.56	4	6.78	6.78	11	19.30	19.30	6	15.38	15.38
	Several times a week	52	31.71	31.71	2	22.22	22.22	20	33.90	33.90	20	35.09	35.09	10	25.64	25.64
	Once a day	44	26.83	26.83	2	22.22	22.22	16	27.12	27.12	11	19.30	19.30	15	38.46	38.46
	Several times a day	4	2.44	2.44	0	0.00	0.00	1	1.69	1.69	1	1.75	1.75	2	5.13	5.13
	No missing responses	164	100	100	9	100	100	59	100	100	57	100	100	39	100	100

[a] % of participants. [b] % of responses.
